# Food Insecurity, Food Based Coping Strategies and Suboptimal Dietary Practices of Adolescents in Jimma Zone Southwest Ethiopia

**DOI:** 10.1371/journal.pone.0057643

**Published:** 2013-03-12

**Authors:** Tefera Belachew, David Lindstrom, Abebe Gebremariam, Dennis Hogan, Carl Lachat, Lieven Huybregts, Patrick Kolsteren

**Affiliations:** 1 Department of Population and Family Health, Jimma University, Jimma, Ethiopia; 2 Department of Food Safety and Food Quality, Ghent University, Ghent, Belgium; 3 Department of Sociology, Brown University, Providence, Rhode Island, United States of America; 4 Nutrition and Child Health Unit, Department of Public Health, Institute of Tropical Medicine, Antwerp, Belgium; University of Ottawa, Canada

## Abstract

Despite the high prevalence of adolescent food insecurity in Ethiopia, there is no study which documented its association with suboptimal dietary practices. The objective of this study is to determine the association between adolescent food insecurity and dietary practices. We used data on 2084 adolescents in the age group of 13–17 years involved in the first round survey of the five year longitudinal family study in Southwest Ethiopia. Adolescents were selected using residence stratified random sampling methods. Food insecurity was measured using scales validated in developing countries. Dietary practices were measured using dietary diversity score, food variety score and frequency of consuming animal source food. Multivariable regression models were used to compare dietary behaviors by food security status after controlling for socio-demographic and economic covariates.

Food insecure adolescents had low dietary diversity score (P<0.001), low mean food variety score (P<0.001) and low frequency of consuming animal source foods (P<0.001). After adjusting for other variables in a multivariable logistic regression model, adolescent food insecurity (P<0.001) and rural residence (P<0.001) were negatively associated with the likelihood of having a diversified diet (P<0.001) and frequency of consuming animal source foods, while a high household income tertile was positively associated. Similarly, multivariable linear regression model showed that adolescent food insecurity was negatively associated with food variety score, while residence in semi-urban areas (P<0.001), in urban areas (P<0.001) and high household income tertile (P = 0.013) were positively associated. Girls were less likely to have diversified diet (P = 0.001) compared with boys.

Our findings suggest that food insecurity has negative consequence on optimal dietary intake of adolescents. Food security interventions should look into ways of targeting adolescents to mitigate these dietary consequences and provide alternative strategies to improve dietary quality of adolescents in Southwest Ethiopia.

## Introduction

Regular intake of adequate quality and quantity diet is vital for optimal health, growth and development of adolescents. In low income countries, suboptimal dietary practices result either from limited access to food supply [1]–[2] or from inadequate knowledge [3] of the importance of a good quality and quantity diet. Inadequate dietary intake related to food insecurity is particularly common. When food supplies are constrained, household members including adolescents are exposed to using coping strategies that compel them to reduce the quality and quantity of foods consumed [4]. Adolescents need diets rich in micronutrients and protein for their pubertal growth spurt. Adolescence is the only time in the life span following infancy when the growth rate is very rapid and individuals acquire a substantial proportion of their adult weight and height [5], and this makes them vulnerable to the consequences of suboptimal diet. However, adolescent nutrition is given less attention in nutrition policies, strategies and programs of low income countries; which might explain the high prevalence of both acute and chronic malnutrition among adolescents in developing countries [6]–[8]. In Ethiopia for instance, adolescents make up about 25% of the total population [9], but, both the national nutrition strategy and the national nutrition program do not identify them as specific target groups [10]–[11].

In Jimma, Ethiopia food insecurity is a common problem [12]. Following an increase in food prices since 2005[13]–[14] and the un-affordability of essential food items especially animal source foods, dietary quality of adolescents is expected to deteriorate further.

Poor quality diet intake during adolescence may lead to malnutrition [13] that could resonate throughout generations as adolescence is sensitive period for development of both future productive and reproductive capacities. It was also reported that food insecurity is associated with an increased morbidity in adolescents [12], [15]–[16], which is hypothesized to be due to poor dietary intake practices.

The available evidence substantiating the association between food insecurity and dietary practices originates from high-income countries [17]–[18] based on household measures of food insecurity as stated by the head of the household. However, household measures of food insecurity may not fully capture the dietary experiences of adolescents [12].

In developing countries where people often share food from a common bowl [19], dietary diversity [20]–[22], food variety [23]–[24] and consumption of animal source foods [25]–[27] are indicators commonly used to assess dietary intake. Adequacy of nutrient intake has been positively associated with the number of different foods consumed [23]–[24], [28]. However, the few studies which assessed the effect of food insecurity on dietary intakes in low income countries used indirect methods based on per capita expenditures on food as reported by the household head rather than direct assessment of the above measures [29]–[31]. These indirect methods may not fully capture the dietary challenges at the individual consumption level. There are no studies that explored the direct association between adolescents’ personal experience of food insecurity and dietary practices, especially in the developing countries’ context where food insecurity is common. This study examined the disparities in dietary diversity, food variety score and frequency of animal source food consumption by adolescent security status.

## Methods

### Study sample

This report is based on data from 2084 adolescents enrolled in the first round of the five year longitudinal study of adolescents conducted in Jimma zone Southwest Ethiopia. The study samples were drawn from urban [Jimma city], semi urban [small towns] and six rural communities “Kebeles” adjacent to the towns to give a representative picture of the area. From the total list of 5795 households generated through census, 3700 households were randomly selected and screened for an eligible adolescent. An adolescent boy or girl in the age group 13–17 years was selected from the households that have this target age group using a Kish[32] resulting in an enrollment of 2100 adolescents for the longitudinal study. The sample size for each study site was allocated based on a probability proportional to size.

Structured household and adolescent questionnaires were used to collect data on food security, socio-demographic and economic variables. The household and adolescent interviews were carried out from August 2005 to February 2006. The questionnaires were interviewer administered and translated to the local languages [Amharic and Oromifa] and their consistency was checked by another person who speaks both languages. The questionnaire for collecting food security, socio-demographic and economic data was pre-tested on 200 adolescents selected from a community in Jimma City that were not included in the main study and the questionnaire was modified based on the pretest observations. The interviewers received one week of intensive training prior to the pre-test and an additional week of training with the final version of the questionnaire before beginning of the actual interviews. Supervisors kept track of the field procedures and checked the completed questionnaires every day to ensure accuracy of the data collected and the research team supervised the data collection team every week.

### Measurements

We used three measures including dietary diversity [20]–[21], food variety [23]–[24] and consumption of animal source foods [25]–[27] to gauge their dietary practices. Dietary diversity [DD] was assessed using a food frequency questionnaire containing 30 food items that are commonly consumed in the study area. The list of food items was developed based on an extensive interview of the data collectors who are from the study area and who knew the culture and language and key informants in the study area on the types of foods commonly consumed. The food frequency questionnaire was pre-tested on 200 adolescents and the food items commonly consumed in the area and the patterns over the week days observed during the pretest were used to refine the food frequency questionnaire. Participants were asked to report the frequency of consumption of each food per day, per week or per month using the past 3 months as a reference [33]. Given the large variation of dietary habits in the local community over the days of the week, the consumption of each food item per day [34] was not taken as a cut-off point to define consumers. Rather, adolescents were coded as a “consumer” of a food item if they had consumed the food item at least once per week [35]. As there is no Ethiopian classification of food groups, the 30 food items [Table 1] of the food frequency questionnaire were grouped into seven groups [gains/vegetables/fruits/dairy/protein foods/oils/Discretionary calories] [Table 2] according to the MyPyramid classification for healthy eating [36]. A Dietary Diversity Score (DDS) was constructed by counting the intake of the food groups over a period of one week [37] based on the definition that it is the sum of food groups consumed over the reference period. For example, an adolescent who consumed one item from each of the food groups at least once during the week would have the maximum DDS of 7. The DDS was converted into tertiles and the highest tertile was used to define “high” dietary diversity score, while the two lower tertiles combined were labeled as “low” dietary diversity score. Food Variety Score [FVS] is the frequency of individual food items consumed in the reference period. It was calculated by counting the consumption of each of the 30 individual food items over the reference period of one week [28], [37] with the maximum FVS to be thirty. The mean FVS were compared by background characteristics and food security status.

**Table 1:** Proportion of adolescents who consumed specific food item at least once in a week in any given week during the last 3 months before the survey by their food security status.*

Food items consumed	Adolescent Food security Status			
	Food secure (%) (n = 1656)	Food insecure (%) (n = 428)	Total (%) (n = 2084)	*P^1^*
***Grains***				
Teff (*Eragrostis abyssinica*)WheatSorghumBarleyMaizeRice	99.486.759.066.779.842.9	98.181.354.951.981.128.7	99.185.658.263.780.040.0	0.0120.0050.126<0.0010.547<0.001
***Vegetables***				
Sweet potatoPotatoCarrotsTomatoCauliflowerGreen leafy vegetables	67.087.750.369.712.781.3	68.087.140.469.29.386.0	67.287.648.369.612.082.3	0.7060.740<0.0010.8140.0540.025
***Fruits***				
AvocadoPapayaBananaPineappleOranges	44.466.791.212.779.4	29.755.684.610.772.7	41.464.489.812.378.0	<0.001<0.001<0.0010.2770.003
***Dairy***				
MilkCheese	57.067.8	42.359.8	54.066.1	<0.0010.002
***Protein Source foods***				
BeefFishChickenGoat or muttonLiverEggsNutsLegumes	32.44.95.726.98.062.468.094.1	20.82.63.016.98.242.551.093.2	30.04.45.124.88.158.364.693.9	<0.0010.0370.027<0.0010.9217<0.001<0.0010.508
***Oils***				
ButterOil	71.498.1	61.997.2	69.497.9	<0.0010.227
***Discretionary Calories***				
Soft drinks	25.4	12.4	22.7	<0.001

Most of the significant differences observed were on food items which became very expensive.

* Percentages refer to the proportion of adolescents who consumed the food item over the last 7 days and are calculated from column total. ^1^The significance level is 0.0016 [0.05/30) due to Bonferroni correction.

A Chi-square test was used to calculate the P values.

**Table 2:** Mean (±SD) intake of food groups per week in any given week during the last 3 months before the survey by food security status and sex.

Food Groups	All (n = 2084)		Boys(n = 1059)		Girls(n = 1025)	
	FS(n = 1656)	FIS(n = 428)	FS(n = 892)	FIS(n = 167)	FS(n = 764)	FIS(n = 261)
Grains	1.00(±0.02)	1.00(±0.05)	1.00(±0.03)	1.00(±0.00)	1.00(±0.00)	1.00(±0.06)
Vegetables	0.99(±0.10)	0.99(±0.08)	0.99(±0.12)	0.99(0.11)	0.99(±0.10)	0.99(±0.06)
Fruits	0.98(±0.12)	0.94(±0.23)***	0.98(±0.13)	0.89(±0.30)***	0.99(±0.11)	0.97(±0.17)
Dairy	0.80(±0.40)	0.71(±0.45)***	0.29(±0.46)	0.12(±0.32)***	0.82(±0.38)	0.69(±0.46)***
Protein source foods(both plant and animal)	1.00(±0.07)	0.99(±0.11)	0.99(±0.09)	0.98(±1.13)	1.00(±0.00)	0.99(±0.09)
Oils	0.99(±0.09)	0.98(±0.14)	0.99(±0.11)	0.97(±0.17)	1.00(±0.04)	0.99(±0.12)
Discretionary calories	0.25(±0.43)	0.12(±0.32)***	0.79(±0.41)	0.74(±0.43)	0.21(±0.41)	0.13(0.33)**

*** P<0.001, **P<0.01.

T-test was done to assess the differences between food secure and food insecure groups by intake of the different food groups. The food items were grouped according to MyPyramid [USDA, 2005).

Means [±SD) are given unless indicated otherwise.

The p values compare the means frequency of consumption of the different food groups per week between food secure and food insecure adolescents and the maximum value for the group is 1.

FS = food Secure, FIS =  Food Insecure.

Animal Source Food [ASF] intake was assessed by summing the number of times each animal source food was consumed over the days of the week. Frequency of ASF consumption was divided into tertiles and the highest tertile was used to define “high” frequency of consumption of ASF, while the two lower tertiles were labeled as “low” frequency of ASF consumption.

We adjusted our analysis for household dependency ratio calculated as the ratio of people who are not expected to be productive [age groups greater than 64 and less than 15 years] to the number of people who are expected to be potentially productive [age 15–64 years]. The dependency ratio was divided in to tertiles. We also adjusted for adolescent educational status categorized based on the current classification the Ministry of Education as “primary” [grade 8 and below] and secondary and above [Grade 9 and above].

Adolescent food insecurity was measured using items adapted from household food insecurity scales that were previously validated for use in developing countries [38]–[40], the details of the methods are described elsewhere[41]. Four items that amply to individual experiences adolescents were used to assess food insecurity. Briefly, adolescents were asked whether in the last three months they (1) had ever worried about having enough food; (2) had to reduce food intake because of shortages of food or money to buy food; (3) had to go without having eaten because of shortage of food or money to buy food and (4) had to ask outside the home for food because of shortage of food or money to buy food. All “Yes” responses were coded “1” and “No” responses were coded “0” and the scores were summed. Adolescents who had food insecurity score of 1 and above were labeled as food insecure. The index has high internal consistency (Cronbach’s Alpha = 0.81).

The study was approved by the Ethical Review Boards of both Brown University (USA) and Jimma University (Ethiopia). Informed verbal consent was obtained both from the parents and each respondent before the interview or measurement as approved by the ethical review committees which followed and documented the study process through supervisory visits.

### Data analysis

The data were entered in double, checked for missing values and outliers and analyzed using SPSS [SPSS Inc. version 16.1., Chicago, Illinois]. First, bivariate analyses were carried out to test differences in DDS and frequency of consumption of ASF by food security status using Pearson’s Chi-square Tests. For FVS, mean differences between groups were analyzed with an independent sample T-test and one way analysis of variance. The mean intake of the different food groups, mean DDS, mean FVS and proportions of adolescent who had a high frequency of ASF intake were computed by adolescent food security status. To identify the predictors of dietary diversity, a multivariable logistic regression model was fitted with DDS as binary dichotomous dependent variables and other covariates. A second multivariable logistic regression was fitted with frequency of ASF consumption as a binary dependent variable. The results are presented as odds ratio (OR) with 95% confidence intervals. For all scenarios models, only variables that showed a significant association (P<0.05) in the bivariate analyses were entered in the adjusted models.

Third, a multivariable linear regression model was fitted to explore associations between the continuous variable FVS and predictors that showed significant association in the bivariate analyses. Normality of the residuals was inspected visually using data QQ-plots and residual-versus-predictor plots for FVS. Bonferroni correction was applied to the level of significance to adjust for multiplicity of tests for food items using (0.05/n). Second degree interaction terms between different predictors were examined and collinearity between variables was checked using variance inflation factor. All tests were two-sided and P<0.05 was considered statistically significant. The results are presented as means and SD and the parameters from the models as β-coefficients and 95% confidence intervals.

## Results

A total of 2084 adolescents were interviewed out of the 2100 planned giving a response rate of 99.2%. Out of these, 1025(49.2%) were girls and 1059(50.8%) were boys. Regarding their residences, 35.8%, 28.3% and 35.9% of adolescents live in the urban, semi-urban and rural areas, respectively. Overall, a total of 428(20.5%) of adolescents had transient food insecurity. Among the food insecure adolescents, larger proportion used a variety of food based coping strategies for a period of 1–7 days during the last three months before the survey, while a small proportion used the coping strategies for greater than 21 days of a month during the past 3 months. Food based coping strategies used by the majority were : reducing the number of meals per day (89.3%), worrying about running out of food (81.8%), spending the whole day without eating (23.8%) and asking for food or money to buy food/begging (20.8%), Table 3.

**Table 3:** Food based coping strategies used among food insecure adolescents in southwest Ethiopia during the last three months before the survey.

Food based coping strategy(n = 428)	Never	1–7 Days	8–21 Days	>21 Days
	%	%	%	%
Number of days adolescent had to reduce the number of meals eaten in a day, because of shortages of food or money to buy food during the last 3 months	10.7	78.0	6.8	4.4
Number of days the adolescent worried that he/she would run out of food or not have enough money to buy food during the last 3 months	18.2	69.6	6.3	5.8
Number of days the adolescent had to spend the whole day without eating, because of shortages of food or money to buy food during the last 3 months	76.2	19.2	1.9	2.8
Number of days the adolescent had to ask for food or money to buy food(beg) during the last 3 months	79.2	17.8	1.4	1.6

The last 3 months refer to time before interview of the adolescent.

Percentage is calculated out of row totals.

The proportion of adolescents who consumed various food items at least once over one week period is presented by their food security status in Table 1. Out of the 30 different food items, there were 20 significant differences in the reported consumption by food secure and food insecure adolescents, although after adjusting for the large number of statistical tests, only 13 differences were significant at the 0.0016 level (0.05/30 tests). Comparing the food secure and food insecure adolescents, a significantly lower proportion of food insecure adolescents consumed micronutrient and protein rich foods such as beef, egg, cheese, chicken, goat/mutton, fish, nuts, dairy products and fruits (P<0.01) compared to their food secure peers.

Table 2 presents the consumption of the seven food groups by adolescent security status and gender. Food insecure adolescents had a significantly lower mean consumption of the dairy products (P<0.001) and fruits (P<0.001) compared to their food secure counterparts for the whole sample. On gender stratified analysis, food insecure boys had a significantly lower mean consumption of dairy products (P<0.001) and fruits (P<0.001) compared to food secure boys. Similarly, food insecure girls had a significantly lower mean consumption of the dairy products (P<0.001) and discretionary calories (P<0.01) compared to food secure girls.

Bivariate analyses of dietary practices by socio-demographic variables and food security status are shown in Table 4. Adolescent food insecurity (P<0.001), high household dependency ratio (P = 0.008) and rural residence (P<0.001) were positively associated with low DDS. Whereas, male gender (P<0.001), age (P<0.001), residence in urban areas (P<0.001) and in semi-urban areas (P<0.001), high household income tertile (P<0.001) and educational status of secondary school or above (P<0.001) were negatively associated with having low DDS. A similar pattern was also observed in the frequency of consumption of ASF.

**Table 4:** Dietary diversity, food variety score and the consumption of animal source food by adolescent and household food security status and socio-demographic variables (n = 2084).

Characteristics	Dietary Diversity Score			FVS		Frequency of ASF Intake*		
	Low (%) (n = 1678)	High (%)† (n = 406)	*p*	Mean(±SD)	*p*	Low (%) (n = 1161)	High (%)† (N = 923)	*P*
Adolescent food securityFood secureFood Insecure	78.090.4	22.09.6	*<0.001*	17.2(±3.9)15.2(±3.8)	*<0.001*	64.772.9	35.327.1	*0.003*
Sex of the adolescentFemaleMale	83.977.2	16.122.8	*<0.001*	16.7(±3.6)16.8(±4.3)	*0.952*	65.267.5	34.832.5	*0.257*
Age in completed years1314151617	87.382.978.175.476.5	12.717.121.924.623.5	*<0.001*	16.3(±3.7)16.9(±3.9)16.7(±4.0)17.1(±4.1)17.1(±4.2)	*0.016*	74.265.665.363.560.9	25.834.434.736.539.1	*<0.001*
Place of residenceUrbanSemi-urbanRural	72.878.390.0	27.221.710.0	*<0.001*	16.7(±4.3)18.0(±3.7)16.0(±3.6)	*<0.001*	49.965.483.6	50.134.616.4	*<0.001*
Household income tertileLowMiddleHigh	87.284.569.9	12.815.530.1	*<0.001*	16.4(±3.8)16.5(±4.0)17.5(±4.0)	*<0.001*	79.271.448.6	20.828.651.4	*<0.001*
Dependency Ratio, TertilesLowMiddleHigh	77.379.984.0	22.720.116.0	*0.008*	17.1(±4.0)16.7(±4.0)16.7(±3.8)	*0.039*	61.665.771.4	38.434.328.6	*0.001*
Educational status of the adolescentPrimarySecondary & above	83.068.4	17.031.6	*<0.001*	16.6(±3.9)17.3(±4.2)	*<0.012*	69.751.4	30.348.6	*<0.001*

† High =  3^rd^ tertile of frequency of ASF consumption, Low =  the two lower tertiles of frequency of ASF consumption combined.

T-test was used for all mea comparisons with two groups and one way-ANOVA was use for comparing more than 2 means.

Proportions were compared using Chi-square tests.

Consumption of ASF refers to consumption of at least one of the 10 ASF in the food frequency questionnaire once per week.

† High  =  DDS of the highest (3^rd^) tertile, Low =  the two lower DDS tertiles combined.

DDS: dietary diversity score, FVS =  food variety score and ASF =  Animal Source foods.

Primary =  grade 1–8.

*Frequency of ASF consumption per week, tertiles: low (<1.3), Middle (3.0), high (4.8).

A multivariable logistic regression analyses carried out to isolate the independent effects of the different covariates on DDS showed that age (OR = 1.15, P = 0.006) and high household income tertiles (OR = 1.61, P = 0.004) were positively associated with having high dietary diversity score, while adolescent food insecurity (OR = 0.42, P<0.001) and residence in the rural area (OR = 0.37, P<0.001) were negatively associated with having high dietary diversity. Similarly, adolescent food insecurity (OR = 0.67, P = 0.003) and rural residence (OR = 0.28, P<0.001) were negatively associated with having high frequency of ASF consumption per week, while high household income tertile (OR = 2.33, P<0.001) and middle household income tertile (OR = 1.32, P = 0.04) were positively associated with having high frequency of consuming ASF (Table 5).

**Table:** **Table 5.** Multivariable logistic regression model predicting the likelihood of having high dietary diversity, high frequency of consuming animal source foods per week among adolescents.^¶^

Predictor Variables(n = 2084)	n	High Dietary diversity Score			High frequency of ASF intake		
		β	OR(95%CI)	*P*	β	OR(95%CI)	*P*
Adolescent food security							
Food secure (ref)	1656		1			1	
Food insecure	428	−0.87	0.42(0.29−0.62)	*<0.001*	−0.41	0.67(0.51−0.87)	*0.003*
Sex of the adolescent							
Male (ref)	1059		1				
Female	1025	−0.41	0.66(0.52−0.84)	*0.001*	—	—	—
Age of the adolescent (years)	2084	0.14	1.15(1.04−1.27)	*0.006*	0.07	1.07(0.99−1.17)	*0.097*
Place of residence							
Urban (ref)	746		1			1	
Semi-urban	589	−0.21	0.81(0.61−1.08)	*0.149*	−0.51	0.60(0.47−0.77)	*<0.001*
Rural	749	−1.00	0.37(0.26−0.53)	*<0.001*	−1.26	0.28(0.21−0.38)	*<0.001*
Household income (tertiles)							
Low(ref)	687		1			1	
Middle	702	0.09	1.09(0.79−1.51)	*0.602*	0.28	1.32(1.01−1.72)	*0.040*
High	695	0.48	1.61(1.16−2.24)	*0.004*	0.85	2.33(1.76−3.08)	*<0.001*
Dependency ratio (tertiles)							
Low (ref)	635		1			1	
Middle	743	0.11	1.12(0.85−1.49)	*0.430*	0.05	1.05(0.83−1.35)	*0.673*
High	706	0.06	1.06(0.78−1.46)	*0.699*	0.10	1.10(0.84−1.44)	*0.487*
Educational status(Adol)							
Primary (ref)	1583		1			1	
Secondary and above	501	0.13	1.14(0.82−1.59)	*0.442*	0.422	1.02(0.76−1.37)	*0.885*
Hosemer Lemeshow Test (P)			0.113			0.895	
Cox and Snell's Pseudo R^2^			0.083			0.071	

Parameters estimates were adjusted for the tabulated variables. Ref: indicates the reference category.

Maximum Variance inflation factor  =  1.96.

¶ Parameters estimates adjusted for: Dependency ratio, household income, gender of the household head.

Coefficients as obtained from a multivariable linear regression model.

High =  3^rd^ tertile for both DDS and frequency of ASF consumption.

Adol =  Adolescent.

In a multivariable linear regression model, adolescent food insecurity (β = −1.99, P<0.001) was negatively associated with FVS, while high household income tertile (β = 0. 64, P = 0.009), residence in urban (β = 0.51, P<0.036) and semi-urban (β =  1.82, P<0.001) areas were positively associated (Table 6).

**Table 6:** Parameter estimates from linear regression predicting food variety score among adolescents.¶

Predictors(n = 2084)	β	SE	*P*
Adolescent food insecurity (Ref. = food secure)	−1.993	0.219	<0.001
Age of adolescent(Years)	0.137	0.072	0.058
Dependency ratio middle tertile (ref: low tertile)	−0.212	0.217	0.328
Dependency ratio tertile high (ref: lowest tertile)	−0.029	0.229	0.898
Household income middle tertile (ref: low tertile)	0.112	0.214	0.601
Household income tertile high (ref: low tertile)	0.636	0.244	0.009
Education: secondary or above (ref: primary)	−0.008	0.271	0.978
Urban residence (ref :Rural)	0.513	0.244	0.036
Semi urban residence (ref: Rural)	1.818	0.232	<0.001

¶ Multivariable linear regression model with the Food Variety Score as a dependent variable and predictors with

P<0.05 of the bivariate model.

Adjusted R^2^ =  0.085.

Ref =  reference category.

Coefficients as obtained from a multivariable linear regression model.

Maximum Variance inflation factor  =  1.78.

SE =  Standard error.

## Discussion

Adolescent food insecurity is prevalent in the study area [12], [42]. We previously reported how food insecurity negatively affects health [12], [16] and educational attainment [41] of adolescents. One of the mechanisms we hypothesized for the above findings was through the negative effect of food insecurity on the dietary practices. This study showed that adolescent food insecurity was an independent predictor of unhealthy dietary practices. After adjusting for various socioeconomic and demographic variables, food insecure adolescents were more likely to have low dietary diversity score, low food variety score and low frequency of animal source food intake. These findings may indicate that food insecure adolescents are at risk of multiple nutrient deficiencies [17], [21], [43]–[44] as a diversified diet containing ASF provides good sources of essential micronutrients like iron, zinc, calcium and vitamins[26]. Studies in both developed [45]–[46] and developing countries [21]–[22], [47]–[48] have shown that DDS is associated with increased nutrient intake and better nutritional status in children. Other studies reported that ASF consumption ensures intake of micronutrients and high quality protein among children in developing countries [25]–[27], [49].

Although ASF contribute significantly (even in small quantities) to dietary quality, their consumption in sub-Saharan African countries is declining due to decreased access and their increased price and due to an increase the population size [49], [50], while it is on the rise in high and middle income countries [27], [49]. This coupled with increasing cost of foods will erode access to ASF among adolescents in low income countries. A study in three developing countries (Bolivia, Burkina Faso, and the Philippines) showed that food secure households had significantly higher total food expenditures as well as expenditures on ASF, vegetables, and fats and oils compared with moderately and severely food-insecure households [29]. A similar study in Bangladesh showed that dietary diversity was associated with per capita food and total expenditure [30].

Food security exists when every individual has access to an adequate quantity and quality food all times [51]. When food supplies are constrained, adolescents may use various copping strategies that might negatively affect their nutrient intake. One of the coping strategies is the reduction in the dietary intake manifested through either reduced number of meals or size of meals [4], [38], [52]–[54]. Poor dietary practices in food constrained situations emanate from the behavioral adaptations in response to food shortage [4], [55]. As reported by Norhasmah et al 2010[4], food related coping strategies follow sequential steps that progress into temporary alteration of the dietary intake patterns. These adaptations are categorized into four themes: food stretching (consuming low quality cheap foods), food rationing (reducing the size or frequency of consumption), food seeking (searching for food in socially unacceptable ways) and food anxiety [4], [38]. Such coping strategies are evident in the lexicon of young people in the study area. These behavioral responses gradually erode the nutrient intakes of food insecure adolescents or those who are members of food insecure households predisposing them to multiple nutrient deficiencies.

Similar to a report from Guatemala [56], our results also show that adolescents in rural areas were less likely to have a diversified diet, high FVS and high frequency of consuming ASF compared to adolescents that live in smaller towns or in the urban areas. This could be due to better awareness [57] and better caring practices [58] of households in urban areas for their children compared with those in rural places, but, more likely this has to do with higher degree of poverty of in the rural areas (as this seems to be consistent with our results).

Our results also showed that girls were less likely to have diversified diet which was similar to the findings of an earlier study in the same cohort [12]. This could probably be due to less access of girls to the declining food resources. Similar bias to boys in the intra-household allocation of food was also reported from other developing countries [59]–[61].

Consumption of a diversified diet, ASF and high FVS were associated with the highest household income tertile. In the face of increasing food practices, household income enhances their capacity to purchase food items [29]–[30], [62] thereby increasing access to good quality diet.

In the absence of interventions, food constraints reflected through having poor dietary practices may lead to negative consequences on the wellbeing of adolescents. As a result, food insecure adolescents may be at risk of morbidity [12], [16] and mortality [63] and poor development[64]–[65]. Preliminary data from this cohort of youth also showed that food insecure youth were at elevated risk of high symptoms of anxiety and depression [Hadley C, Emory University, pers. comm.].

In Ethiopia, the government is developing an accelerated stunting reduction strategy which is mainly focusing on food based approaches. Evidence shows that animal ownership has potential for economic benefits and contribution to ASF consumption in poor communities [25]. There is a need for innovative ways of enhancing both local production and increased consumption of ASF by adolescents and growing children at the household level. A report from the highlands of Ethiopia showed that an integrated approach for the promotion of local dairy goats managed by women “..*Through a combination of better management techniques, genetic improvements"* lead to better access of households to ASF [66]. As in many other developing countries, adolescents make large segment of the population in Ethiopia. Given the fact that food insecurity is a major problem of the country and that adolescence is the second period of human life cycle when a very rapid growth, development with maturation of different organs occurs, the findings call for attention. A poor dietary practice that occurs at this age may lead to malnutrition that perpetuates itself [67].

We used personal experiences of adolescent food insecurity to demonstrate its direct consequence on their dietary practices rather than household food insecurity reported by the household head. A recent study also showed adults’ responses do not represent children’s food security experience as food insecurity in children may be grounded in the intra-household resource distribution dynamics which are beyond the immediate lack of food that adults are aware of [68].

We used a food frequency questionnaire to assess the dietary intake. Although the food frequency questionnaire was developed by the investigators who are residents of the study area with the help of field workers who were well acquainted with the local culture based on the pretest results, the fact that the questionnaire is not validated is the limitation of this study. As there is within individual variability of dietary intake in the study area over the days of the week, we based our definition of a consumer on a reference period of one week. People who might have consumed a food item more than once per week were also categorized with those who consumed only once per week which might underestimate the quantity consumed. Dietary practice was based on the qualitative measures of DDS, FVS and consumption of ASF. Addition of portion sizes would have improved our ability to typify the quantity of the diet the youth consumed [47]. However, quantitative assessment of nutrient intake is very difficult in contexts where people consume from a common bowl [69] as is common throughout Ethiopia. Designing dietary assessment methods that are valid in populations that consume from a common bowl or share food from the same plate [19] in the Ethiopian context is a challenge that requires additional research.

Our study has implication for the current global food crisis. Although studies show that adult members of the household buffer children from food insecurity [12], [70], the recent massive increase in the price of food items both globally [14], [71] and in Ethiopia [14], [72] would compromise the availability of important food items at household level [29]–[30] inducing poor quality diets among adolescents. It was also reported that children were not always protected from food insecurity, especially when the head of the family was unemployed and was extremely or abuses substance [73] and that the relationship between food insecurity in the children-women subunit also varied depending the status of the woman in the family [74].

In conclusion, food insecurity has significant negative consequence on the diets of adolescents in the southwest Ethiopia that puts them at risk of developing multiple nutrient deficiencies. As food insecurity is a frequent problem and adolescents constitute a large segment of the population in Ethiopia, the findings indicate the need for programs to improve adolescent nutrition. Food security interventions should look into ways of enhancing both diversified local production and consumption of nutrient rich foods to prevent food insecurity and its nutritional consequences in adolescents.

## References

[1] Leyna GH, Mmbaga EJ, Mnyika KS, Hussain A, Klepp KI (2010) Food insecurity is associated with food consumption patterns and anthropometric measures but not serum micronutrient levels in adults in rural Tanzania. Public Health Nutr 13(9): 1438–44. 20196915 10.1017/S1368980010000327

[2] Becquey E, Martin-Prevel Y, Traissac P, Dembélé B, Bambara A, et al. (2010 Dec)The household food insecurity access scale and an index-member dietary diversity score contribute valid and complementary information on household food insecurity in an urban West-African setting. J Nutr 140(12): 2233–40. 20962154 10.3945/jn.110.125716

[3] Oldewage-Theron WH, Egal AA (2010) Nutrition knowledge and nutritional status of primary school children in QwaQwa. S Afr J Clin Nutr 23(3): 149–154.

[4] Norhasmah S, Zalilah MS, Mohd Nasir MT, Kandiah M, Asnarulkhadi AS (2010) A qualitative study on coping strategies among women from food insecurity households in Selangor and Negeri Sembilan. Mal J Nutr 16(1): 39–54. 22691852

[5] Rogol AD, Clark PA, Roemmich JN (2000) Growth and pubertal development in children and adolescents: effects of diet and physical activity. Am J Clin Nutr 72(2 Suppl): 521S–8S. 10919954 10.1093/ajcn/72.2.521S

[6] Alam N, Roy SK, Ahmed T, Ahmed AM (2010) Nutritional status, dietary intake, and relevant knowledge of adolescent girls in rural Bangladesh. J Health Popul Nutr 28(1): 86–94. 20214090 10.3329/jhpn.v28i1.4527 PMC2975850

[7] Jackson M, Samms-Vaughan M, Ashley D (2002) Nutritional status of 11–12-year-old Jamaican children: coexistence of under and over nutrition in early adolescence. Public Health Nutrition 5(2): 281–288. 12020379 10.1079/PHN2002262

[8] Mulugeta A, Hagos F, Stoecker B, Kruseman G, Linderhof V, et al (2009) Nutritional status of adolescent girls from rural communities of Tigray, Northern Ethiopia. Ethiop J Health Dev 23(1): 5–11.

[9] Federal Democratic Republic of Ethiopia Ministry of Health (2003) Adolescent reproductive health extension package, Addis Ababa. Available: http://cnhde.ei.columbia.edu/training/documents/Adolescence.pdf. Accessed 2011 Apr 10.

[10] Federal Ministry of Health (April 2008) National Nutrition Program of Ethiopia, Program Implementation Manual (NNP) – I.

[11] Federal Ministry of Health (Jan 2008) National Nutrition Strategy of Ethiopia. Available: http://www.iycn.org/files/EthiopiaNationalNutritionStrategy.pdf. Accessed 2011 Mar 10.

[12] Hadley C, Lindstrom D, Tessema F, Belachew T (2008) Gender bias in food insecurity experiences of adolescents in Jimma Zone. Soc Sci Med 66: 427–438. 17931763 10.1016/j.socscimed.2007.08.025 PMC2791354

[13] Brinkman HJ, de Pee S, Sanogo I, Subran L, Bloem MW (2010) High food prices and the global financial crisis have reduced access to nutritious food and worsened nutritional status and health. J Nutr 140(1): 153S–61S. 19939996 10.3945/jn.109.110767

[14] Sanogo I (2009) Global food price crisis and household hunger: a review of recent food security assessments findings. Humanitarian Exchange Magazine. Available: http://www.odihpn.org/report.asp?id=2988. Accessed 2011 Mar 10.

[15] Pan Y, Pratt CA (2008) Metabolic syndrome and its association with diet and physical activity in US adolescents. J Am Diet Assoc 108(2): 276–86. 18237576 10.1016/j.jada.2007.10.049

[16] Belachew T, Haley C, Lindtsrom D, Gebremariam A, Getachew Y, et al (2011) Gender differences in food insecurity and morbidity among adolescents in southwest Ethiopia. Pediatrics 127(2): e397–404. 10.1542/peds.2010-0944 PMC3387862 21220395

[17] Kirkpatrick SI, Tarasuk V (2008) Food insecurity is associated with nutrient inadequacies among Canadian adults and adolescents. J Nutr 138(3): 604–12. 18287374 10.1093/jn/138.3.604

[18] Duffy P, Zizza C, Jacoby J, Tayie FA (2009) Diet quality is low among female food pantry clients in Eastern Alabama. J Nutr Educ Behav 41(6): 414–9. 19879497 10.1016/j.jneb.2008.09.002

[19] Hudson GJ (1995) Food intake in a West African village: estimation of food intake from a shared bowl. Br J Nutr 73(4): 551–569. 7794871 10.1079/bjn19950058

[20] Kennedy G, Fanou-Fogny N, Seghieri C, Arimond M, Koreissi Y, et al (2010) Food groups associated with a composite measure of probability of adequate intake of 11 micronutrients in the diets of women in urban Mali. J Nutr ;140 (11): 2070S–8S. 10.3945/jn.110.123612 20881080

[21] Kennedy GL, Pedro MR, Seghieri C, Nantel G, Brouwer ID (2007) Dietary diversity is a useful indicator of micronutrient intake in non-breastfeeding Filipino children. J Nutr 137(2): 472–7. 17237329 10.1093/jn/137.2.472

[22] Arimond M, Wiesmann D, Becquey E, Carriquiry A, Daniels MC, et al (2010) Simple food group diversity indicators predict micronutrient adequacy of women's diets in 5 diverse, resource-poor settings. J Nutr 140(11): 2059S–69S. 20881077 10.3945/jn.110.123414 PMC2955880

[23] Ogle BM, Hung PH, Tuyet HT (2001) Significance of wild vegetables in micronutrient intakes of women in Vietnam: an analysis of food variety. Asia Pac J Clin Nutr 10(1): 21–30. 11708605 10.1046/j.1440-6047.2001.00206.x

[24] Marshall TA, Stumbo PJ, Warren JJ, Xie XJ (2001) Inadequate nutrient intakes are common and are associated with low diet variety in rural, community-dwelling elderly. J Nutr 131(8): 2192–6. 11481416 10.1093/jn/131.8.2192

[25] Allen LH (2003) Interventions for micronutrient deficiency control in developing countries: past, present and future. J Nutr 133(11 Suppl 2)3875S–3878S. 10.1093/jn/133.11.3875S 14672284

[26] Neumann C, Harrisa DM, Rogers LM (2002) Contribution of animal source foods in improving diet quality and function in children in the developing world. Nutrition Research 22(1): 193–220.

[27] Hop LT (2003) Programs to improve production and consumption of animal source foods and malnutrition in Vietnam. J Nutr 133(11 Suppl 2)4006S–4009S. 14672303 10.1093/jn/133.11.4006S

[28] Torheim LE, Barikmo I, Parr CL, Hatl A, Ouattara F, et al (2003) Validation of food variety as an indicator of diet quality assessed with a food frequency questionnaire for Western Mali. Eur J Clin Nutr 57(10): 1283–91. 14506490 10.1038/sj.ejcn.1601686

[29] Melgar-Quinonez HR, Zubieta AC, MkNelly B, Nteziyaremye A, Gerardo MF, et al (2006) Household food insecurity and food expenditure in Bolivia, Burkina Faso, and the Philippines. J Nutr 136(5): 1431S–1437S. 16614440 10.1093/jn/136.5.1431S

[30] Thorne-Lyman AL, Valpiani N, Sun K, Semba RD, Klotz CL, et al (2010) Household dietary diversity and food expenditures are closely linked in rural Bangladesh increasing the risk of malnutrition due to the financial crisis. J Nutr 140(1): 182S–8S. 19923385 10.3945/jn.109.110809

[31] Pérez-Escamilla R, Segall-Corrêa AM, Maranha LK, Sampaio M de F, Marín-León L, et al (2004) An adapted version of the U.S. Department of Agriculture Food Insecurity Module is a valid tool for assessing household food insecurity in Campinas, Brazil. J Nutr 134(8): 1923–8. 15284377 10.1093/jn/134.8.1923

[32] Kish L (1949) A procedure for objective respondent selection within the household. Jam Stat Assoc (44): 380–387.

[33] Rodrıguez MM, Mendez H, Torun B, Schroeder D, Stein DA (2002) Validation of a semi-quantitative food-frequency questionnaire for use among adults in Guatemala. Public Health Nutr 5(5): 691–698. 12372164 10.1079/PHN2002333

[34] Azadbakht L, Mirmiran P, Azizi F (2005) Variety scores of food groups contribute to the specific nutrient adequacy in Tehranian men. Eur J Clin Nutr 59(11): 1233–40. 16015253 10.1038/sj.ejcn.1602234

[35] Kant AIL, Thompson FE (1997) Measures of overall diet quality from a food frequency questionnaire: National Health Interview Survey, 1992. Nutr Res 17(9): 1443–1456.

[36] US Deparment of Agriculture, Human Nutrition Information Service (2005) My pyramid. Home Garden Bulletin. Washington DC: US Department of Agriculture.

[37] Torheim LE, Ouattara F, Diarra M, Thiam F, Barikmo I, et al (2004) Nutrient adequacy and dietary diversity in rural Mali: association and determinants. Eur J Clin Nutr 58(4): 594–604. 15042127 10.1038/sj.ejcn.1601853

[38] Coates J, Frongillo EA, Rogers BL, Webb P, Wilde PE, et al (2006) Commonalities in the experience of household food insecurity across cultures: what are measures missing? J Nutr 136(5): 1438S–1448S. 16614441 10.1093/jn/136.5.1438S

[39] Coates J, Frongillo EA, Rogers BL, Swindale A, Bilinsky P (2006) Measuring household food insecurity: why it’s so important and yet so difficult to do? J Nutr 136(5): 1404S–1408S. 16614437 10.1093/jn/136.5.1404S

[40] Frongillo EA, Nanama S (2006) Development and validation of an experience-based measure of household food security within and across seasons in northern Burkina Faso. J Nutr 136(5): 1409S–1419S. 16614438 10.1093/jn/136.5.1409S

[41] Belachew T, Hadley C, Lindstrom D, Gebremariam A, Lachat C, et al (2011) Food insecurity, school absenteeism and educational attainment: a longitudinal study. Nutrition J 10: 29. 21477343 10.1186/1475-2891-10-29 PMC3090325

[42] Hadley C, Belachew T, Lindstrom D, Tessema F (2009) The forgotten population? Youth, food insecurity, and rising prices: implications for the global food crisis. NAPA Bull 32: 77–91. 24489524 10.1111/j.1556-4797.2009.01029.x PMC3907938

[43] Moursi MM, Arimond M, Dewey KG, Trèche S, Ruel MT, et al (2008) Dietary diversity is a good predictor of the micronutrient density of the diet of 6- to 23-month old children in Madagascar. J Nutr 138(12): 2448–53. 19022971 10.3945/jn.108.093971

[44] Steyn NP, Nel JH, Nantel G, Kennedy G, Labadarios D (2006) Food variety and dietary diversity scores in children: are they good indicators of dietary adequacy? Public Health Nutr 9(5): 644–50. 16923296 10.1079/phn2005912

[45] Kant AK (2004) Dietary Patterns and Health Outcomes. J Am Diet Assoc 104: 615–635. 15054348 10.1016/j.jada.2004.01.010

[46] Nicklas TA, Baranowski T, Cullen KW, Berenson G (2001) Eating patterns, dietary quality and obesity. J Am Coll Nutr 20(6): 599–608. 11771675 10.1080/07315724.2001.10719064

[47] Daniels MC, Adair L, Popkin BM, Truong Y (2009) Dietary diversity scores can be improved through the use of portion requirements: an analysis in young Filipino children. Eur J Clin Nutr 63(2): 199–208. 17971828 10.1038/sj.ejcn.1602927

[48] Onyango AW (2003) Dietary diversity, child nutrition and health in contemporary African communities. Comp Biochem Physiol A Mol Integr Physiol 136(1): 61–69. 14527630 10.1016/s1095-6433(03)00071-0

[49] Speedy AW (2003) Global production and consumption of animal source foods. J Nutr 133: 4048S–4053S. 14672310 10.1093/jn/133.11.4048S

[50] Fram MS, Frongillo EA, Jones SJ, Williams RC, Burke MP, et al (2011) Children are aware of food insecurity and take responsibility for managing food resources. J Nutr 141(6): 1114–9. 21525257 10.3945/jn.110.135988

[51] Larsen CS (2003) Animal source foods and human health during evolution. J Nutr 133(11): 38935–38975. 10.1093/jn/133.11.3893S 14672287

[52] FAO (2009) State of food insecurity in the world: Economic crisis impacts and lessons leaned. Available:ftp://ftp.fao.org/docrep/fao/012/i0876e/i0876e.pdf. Accessed 2011 Apr 1.

[53] Ibrahim H, Uba-Eze NR, Oyewole SO, Onuk EG (2009) Food security among urban households: a case study of Gwagwalada area council of the federal capital territory Abuja, Nigeria. PJN (6): 810–813.

[54] Maxwell D, Watkins B, Wheeler R, Collins G (Sept 2003) The coping strategies index: A tool for rapidly measuring food security and the impact of food aid programmes in emergencies, FAO. Available: http://www.fao.org/crisisandhunger/root/pdf/ maxwell.pdf. Accessed 2011 Mar 16.

[55] Mammen S, Bauer JW, Richards L (2009) Understanding persistent food insecurity: a paradox of place and circumstance. Soc Indic Res 92: 151–168.

[56] Hamelin A-M, Habicht J-P, Beaudry M (1999) Food Insecurity: consequences for the household and broader social implications. J Nutr 129: 525S–528S. 10064323 10.1093/jn/129.2.525S

[57] Alarcón JA, Adrino FJ (1991) Urban-rural differences in food intake of poor families in Guatemala. Arch Latinoam Nutr Sep 41(3): 327–35. 1824512

[58] Woodward DR; Cumming FJ (2000) Ball PJ (2000) Williams HM (2000) Hornsby H (2000) et al Urban-rural differences in dietary habits and influences among Australian adolescents. Ecology of Food and Nutrition 39(4): 271–292.

[59] Smith LC, Ruel MT, Ndiaye A (2005) Why is child malnutrition lower in urban than in rural areas? Evidence from 36 developing countries. World Development 33(8): 1285–1305.

[60] Senauer B, Garcia M, Jacinto E (1988) Determinants of the intra-household allocation of food in the rural Philippines. Am J Agric Econ; 70 (1): 170–180.

[61] Behrman J (1988) Intra-household allocation of nutrients in Rural India: are boys favored? Do parents exhibit inequality aversion? Oxf Econ Pap 40(1): 32–54.

[62] Frongillo EA Jr, Begin F (1993) Gender bias in food intake favors male preschool Guatemalan children. J Nutr 123(2): 189–196. 8429367 10.1093/jn/123.2.189

[63] Christian P (2010) Impact of the economic crisis and increase in food prices on child mortality: exploring nutritional pathways. J Nutr; 140 (1): 177S–181S. 10.3945/jn.109.111708 PMC2793127 19923384

[64] Belsky DW, Moffitt TE, Arseneault L, Melchior M, Caspi A (2010) Context and sequelae of food insecurity in children’s development. Am J Epidemiol 172(7): 809–18. 20716700 10.1093/aje/kwq201 PMC2984258

[65] Cook JT, Frank DA (2008) Food security, poverty, and human development in the United States. Ann N Y Acad Sci 1136: 193–209. 17954670 10.1196/annals.1425.001

[66] Ayele Z, Peacock C (2003) Improving access to and consumption of animal source foods in rural households: the experiences of a women-focused goat development program in the highlands of Ethiopia. J Nutr; 133 (11 Suppl 2)3981S–3986S. 10.1093/jn/133.11.3981S 14672299

[67] Darnton-Hill I, Nishida C, James WP (2004) A life course approach to diet, nutrition and the prevention of chronic diseases. Public Health Nutrition 7(1A): 101–121. 14972056 10.1079/phn2003584

[68] Fram MS, Frongillo EA, Jones SJ, Williams RC, Burke MP, et al (2011) Children are aware of food insecurity and take responsibility for managing food resource . J Nutr 141(6): 1114–9. 21525257 10.3945/jn.110.135988

[69] Teufel NI (1997) Development of culturally competent food-frequency questionnaire. Am J Clin Nutr 65(suppl): 1173S–1178S. 9094917 10.1093/ajcn/65.4.1173S

[70] Kuku O, Gundersen C, Garasky S (2011) Differences in food insecurity between adults and children in Zimbabwe. Food Policy 36(2): 311–317.

[71] Cohen MJ, Garrett JL (2009) The food price crisis and urban food [in) security. Human settlements working paper series, UNFPA, IIED. Available from: http://pubs.iied.org/pdfs/ 10574IIED.pdf. Accessed 2011 Mar 16.

[72] WFP & UNICEF (2009) Summary of food security and vulnerability in selected urban centers of Ethiopia, August 2009. Available: http:// home.wfp.org/stellent/groups/public/ documents/ena/ wfp221386.pdf. Accessed 2011 Mar 16.

[73] Bernal J, Frongillo EA, Herrera H, Rivera J (2012) Children live, feel, and respond to experiences of food insecurity that compromise their development and weight status in peri-urban Venezuela. J Nutr 142(7): 1343–9. 22623397 10.3945/jn.112.158063

[74] Nanama S, Frongillo EA (2012) Women’s rank modifies the relationship between household and women’s food insecurity in complex households in northern Burkina Faso. Food Policy 37(3): 217–225.

